# Maternal gastric carcinoma with metastasis to the placenta: A case report

**DOI:** 10.3892/ol.2014.2529

**Published:** 2014-09-12

**Authors:** YUXUAN CHEN, YAO LI, HONG WANG, JUNLI LU, MULAN JIN, ZHENYU ZHANG

**Affiliations:** Department of Obstetrics and Gynecology, Beijing Chaoyang Hospital, Affiliated Hospital of Capital Medical University, Beijing 100020, P.R. China

**Keywords:** gastric cancer, pregnancy, metastasis, placenta

## Abstract

In the current report a case of maternal gastric carcinoma with metastasis to the placenta is presented. Gastric cancer is a worldwide health issue, which accounts for ~8.6% of newly diagnosed cancer cases annually. However, the simultaneous occurrence of gastric cancer and pregnancy is rare; to date, only 0.1% of all cases of gastric cancer occur during pregnancy. Furthermore, among all cases of gestational gastric cancer, only six cases have been reported to exhibit metastasis to the placenta or fetus. In the current study another such case is presented. Based on the results of previous studies, further discussion regarding the diagnosis and prognosis of gastric cancer during pregnancy in the mother and fetus is presented.

## Introduction

Gastric cancer presents a worldwide health issue, which accounts for ~934,000 (8.6%) of newly diagnosed cancer cases and 700,349 mortalities annually. Almost two-thirds of cases occur in Eastern Europe, South America and Asia, with 42% of cases occurring in China alone (1). The mean age of gastric carcinoma patients is 60 years, with a relatively infrequent occurrence in individuals aged <40 years. In addition, gastric cancer is more common in males than females, with a ratio of 1.7:1 (2). Only 0.1% of all cases of gastric carcinoma occur during pregnancy (3). In Japan, the rate of gastric cancer during pregnancy is reported to be just 0.016% (4); however, in China, it exhibits a frequency of 0.67%, which is significantly higher than that of other countries (5). The diagnosis of this type of cancer is difficult due to the high frequency of benign gastrointestinal symptoms that are presented during normal pregnancies, which results in a poor prognosis, resulting in 88% of females succumbing to the disease within one year (6).

Gastric cancer metastasis invade the surrounding tissues and distant organs, affecting cancer cell motility, intravasation, transit in the blood or lymph and extravasation. Friedreich (7) reported the first case of carcinoma metastasis to the fetus in 1866; however, metastasis to the placenta or fetus is extremely rare with <100 cases reported (8). The most common among these are malignant melanomas, accounting for 30% of cases. Lung, hematological and breast malignancies are the second most common malignancies involved and gastric cancer accounts for <5% of the cases reported (3,9). To date, to the best of our knowledge, only six cases of metastasis from gastric cancer to the placenta or fetus have been reported. In the current study the seventh case is presented, which was the first case identified in China. The patient provided written informed consent.

## Case report

A 35-year-old female (gravida, 2; para, 0) was admitted to Beijing Chaoyang Hospital (Beijing, China) at 34 weeks of gestation and was diagnosed with preeclampsia due to high blood pressure and a recent occurrence of proteinuria. The patient experienced occasional nausea and epigastric pain during the second trimester, however, did not receive adequate medical treatment.

Physical examination revealed that the uterus was normal for the gestational stage, without abdominal tenderness or peritoneal symptoms. In addition, the generalized superficial lymph nodes were found to be of normal size.

Laboratory test results revealed a white blood cell count of 13.94×10^9^ cells/l, a red blood cell count of 2.86×10^12^ cells/l, a hemoglobin level of 94 g/l, a platelet count of 125×10^9^ cells/l, a D-Dimer level of 22.55 mg/l, a fasting blood glucose (Fbg) level of 121.1 mg/dl, aspartate aminotransferase levels of 14 U/l, alanine aminotransferase levels of 13 U/l and albumin levels of 27.6 g/l.

An abdominal ultrasound revealed a 34-week single pregnancy. Two days later the patient developed a coagulation disorder, which resulted in a decrease in serum Fbg to 94.3 mg/dl. An emergency cesarean section under spinal anesthesia was performed. The surgery revealed chylous ascites (100 ml) and the placenta was pathologically analyzed. A premature female infant (weight, 2,210 g) with an Apgar score (10) of 10 at 1, 5 and 10 min, respectively, was delivered in a stable condition and was transferred to the neonatal intensive care unit of Beijing Chaoyang Hospital.

**Figure 1:**
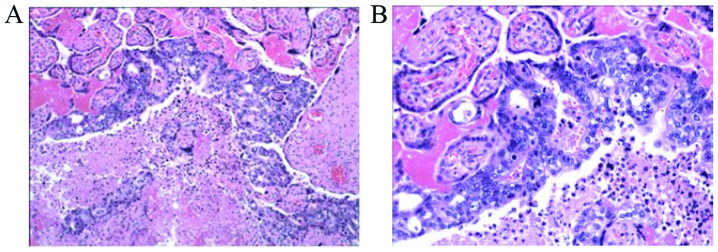
Microscopic examination of the placenta revealed gastric primary malignant cells infiltrating the intervillous space and villous. (A) Magnification ×100. (B) Magnification, ×400.

Macroscopic examination of the placenta revealed no gross metastasis. However, microscopic analysis revealed gastric primary malignant cells within the intervillous space with villous invasion (Fig. 1). The umbilical cord and membranes appeared normal. Computed tomography scan revealed fundus thickening and enlarged retroperitoneal lymph nodes. Gastroendoscopy was performed and revealed linitis plastica with hypertrophic rugae and ulceration. In addition, a gastric biopsy revealed an intermediate differentiated gastric adenocarcinoma. The patient survived and has received chemotherapy. Furthermore, the infant has been reported to be well six months following delivery, with no evidence of fetal metastasis.

## Discussion

There is often a delay in the diagnosis of gastric cancer during pregnancy as the digestive symptoms are common and commonly considered to be a benign response that is induced by the pregnancy itself. This delay in diagnosis results in a poor prognosis resulting in 88% of females succumbing to the disease within one year (6).

Of the 92 cases of gastric cancer reviewed by Jaspers *et al* (11), almost all were found to be at an advanced stage of gastric cancer with only two cases diagnosed with early stage cancer. Early gastric cancer is difficult to detect during pregnancy, as the digestive symptoms of pregnancy, including nausea and vomiting, are common and do not usually require medical attention. Yoshida *et al* (4) reported a case of a patient with vomiting and epigastric pain in the second trimester. The obstetricians diagnosed the patient with early stage gastric cancer, which may have facilitated a favorable prognosis for the mother and infant. Thus, patients with unusual digestive symptoms subsequent to the first trimester require examination. Gastroendoscopy has been identified as an effective method for the detection of early gastric cancer and is regarded as a relatively safe procedure that may be performed, when clinically required, during gestation (12,13).

Gastric carcinoma diagnosed in pregnancy is rare, occurring in only 0.1% of all cases of gastric carcinoma. Maternal malignancy with metastasis to the placenta or fetus is extremely rare with <100 cases reported, of which only ~20% cases were reported to exhibit fetal metastasis (3). Gastric carcinoma accounts for <5% of the reported cases. The majority of the reported cases were identified in the intervillous space without villous invasion; villous invasion is a significant risk factor for fetal involvement, which was present in the current case. Previous studies have recommended that a close follow-up of the healthy infant should be performed, including a physical examination, chest X-ray and liver function tests every six-months for the first two years of life. For cases exhibiting placental and fetal metastasis, further immunohistochemical examination of the mother and fetus is also advised (12).

In conclusion, the potential of gastric cancer during pregnancy must not be ignored, particularly in females exhibiting unusual digestive symptoms. Further investigation must be performed promptly for such patients, as early detection and intervention are essential for improving the prognosis of the mother and fetus. Histopathological examination of the placenta with precise examination of the intravillous spaces and villous is considered to be essential in every case of malignancy during pregnancy.
